# Supplementary material for: Woman authorship in pre-print versus peer-reviewed oral health-related publications: A two-year observational study
Source: PLoS One. 2021 Dec 6;16(12):e0260791. doi: 10.1371/journal.pone.0260791 (PMC8648106; doi:10.1371/journal.pone.0260791)
Supplement: Attachment — Submitted filename: PLOSONE letter of response.docx [file pone.0260791.s002.docx]

10/24/21

Dear Editor,

We would, first, thank the reviewers for the constructive review and comments. We have revised the manuscript based on the reviewers’ comments. Below is a point-by-point summary of edits with their location in the manuscript. All changes in the manuscript are highlighted.

Reviewer 1

Comment 1. *Financial disclosure statement not very detailed - what I assume is a grant number is provided, but there is no link to a URL or further details.*

We have clarified on the financial disclosure as required by the journal

Comment 2. *I could not see a link to information to support the data underlying the results*

We have added a supplemental file with the data set

Comment 3. *At line 125, an indication of the gender composition in dental academia in the US would be informative.*

We have revised the text accordingly.

Comment 4. *Regarding gender assignation at line 178 - was this based on first name?*

The text is revised based on this comment

Comment 5. *I was a little confused in lines 209 - 216 and feel this may need to be expressed more clearly: I read it as 4 peer review publications excluded based on single authors (in which case 71 - 4 is 67 publications in final analysis)? Why were 2 pre-prints excluded?*

We have revised the manuscript based on the comment above and clarified on the exclusions.

Comment 6. *I don't know a lot about statistics, but found the tables and graphs very readable*

Thank you for the comment.

Comment 7. *I found the discussion and conclusion very informative, but am not clear about how a claim of increased productivity can be made given the collection of data at one time point (line 426) and lack of data on pre-print trends in oral health (line 409).*

The manuscript was revised based on the comment.

Further, in terms of the additional journal requirements:

1. We reviewed the manuscript style based on the submission guidelines.
2. We have added the statement that “the funders had no role in study design, data collection and analysis, decision to publish or preparation of the manuscript”.
3. We have added a supplemental file with the research data set to facilitate replication studies.
4. We included Tables 1 and 4 in the manuscript body and connected to the text.
5. We confirmed the accuracy of the references.

Thank you in advance

Effie Ioannidou
